# Historical Sketch of the Mississippi Valley Association of Dental Surgeons

**Published:** 1887-03

**Authors:** 


### Historical Sketch of the Mississippi Valley Association of Dental Surgeons.

AN ADDRESS DELIVERED AT THE FORTY-THIRD ANNUAL MEETING BY THE PRESIDENT, E. G. BETTY, D.D.S.

It has always seemed proper to me that, some one capable of the task, should undertake the writing of an historical sketch of this, the oldest existing dental society in the world, and I am only sorry that it has not been done by one of those who were instrumental in its organization, for, he would have at his command, a memory well-stored with rich anecdote which cannot now be extracted from the almost faded minutes in this book before you.

Almost half a century of busy years have passed away since the first gleam of light shed its rays upon the waste of waters, and what was then not much more than a mixture of the callings of the barber and the jeweller, is now become a distinct and noble profession, having for its aim, the conservation and integrity of an important part of the economy, of the noblest work of God.

Since this Society was formed the world, in many ways, has made vast progress in all directions, to instance which and to contrast the then and now, it is sufficient to mention the telegraph, anæsthesia, the cylinder press, and the telephone. In dentistry, the founding of colleges, the building up of a large literature in its several forms, the burring engine, rubber-cloth, and cohesive gold, leaving out of the account the part dentistry took in the discovery of anæsthesia.

However, it is not my intention to occupy time with a long preamble, or I might continue in this strain for an indefinite period, rehearsing facts that all are more or less familiar with, so I will proceed at once with our subject, doing the best I can to interpret the records in the minute book.

On page number one, we are confronted with the statement that, "In compliance with a call from the 'Cincinnati Association of Surgeon Dentists,' in connection with other members of the profession in the West, a convention of professional dentists met in Cincinnati on Tuesday, the 13th of August, 1844, at 11 o'clock A. M, in the lecture room of the Medical College of Ohio."

There were present at this meeting the following gentlemen: Joseph Taylor, Maysville, Ky.; J. P. Ullery, Lawrenceburg, Ind.; A. D. Bigelow, Newark, O.; James Clark, Lebanon, O.; D. P. Hunt, Indianapolis, Ind.; G. D. Teter, Ripley, O.; D. B. Wheeler, Xenia, O.; Wm. B. Ross, Newport, Ky.; F. E. Suire, Madison, Ind.; J. W. Cook, P. Knowlton, Cincinnati, O.; A. Berry, Raymond, Miss.; J. B. Ross, Philadelphia, Pa.; M. Rogers, John Allen, H. Crane, Wm. M. Hunter, W. J. Madeira, Chas. Bonsall, Jas. Taylor, Cincinnati, O.

In addition to these gentlemen, who were present in person, there were present by proxy: H. Thompson, Columbus, O.; W. Bashan, Dayton, O.; B. Strickland, Cleveland, O.; S. P. Hullihen, Wheeling, Va.; W. H. Goddard, Louisville, Ky.; Wm. R. Winton, Dayton, Ind. (?); B B. Brown, St. Louis, Mo.; W. E. Ide, Zanesville, O.

A committee of five was nominated to draft a constitution, Drs. Allen, Cook, Rogers, Bigelow, and Hunt, being the gentlemen chosen. They presented a constitution (which was adopted), preceded by the following preamble, which, by the way, has rarely, if ever, had its sentiment improved upon by any society since organized :

"The undersigned, practical dentists of the Mississippi Valley, deem it expedient to form an association for the purpose of mutual improvement in the science and practice of our profession. Desirous of promoting the exercise of that gentlemanly courtesy which should characterize members of liberal professions in both social and professional intercourse, believing also that by frequent interchange of opinions and observations in practice, by reporting from time to time cases of interest as they occur in individual practice, we may do much to elevate the character and standing of our profession, and make it worthy the confidence of an enlightened public."

Then follows the Constitution, which, I believe, has been reconstructed, but never definitely acted upon, so that the Society to-day is practically working without one, "a consummation devoutly to be wished," as such an instrument is by some deemed hurtful to the practical working of a scientific body; why, I do not know.

The final organization of the Society was completed by the election of officers, as follows:

President, Jesse W. Cook, Cincinnati, O.

First Vice President, A. D. Bigelow, Newark, O.

Second Vice President, Joseph Taylor, Maysville, Ky.

Third Vice President, D. P. Hunt, Indianapolis, Ind.

Recording Secretary, W. B. Ross, Newport, Ky.

Corresponding Secretary, James Taylor, Cincinnati, O.

Treasurer, Charles Bonsall, Cincinnati, O.

Executive Committee, M. Rogers, John Allen, Cincinnati, O.; F. E. Suire, Madison, Ind.

The first address or essay delivered before the newly organized Society was by the honored and never-to-be-forgotten James Taylor, his subject being Medico-Dental Education. The address was, on motion, adopted by the Society and ordered filed among its archives, but which, I am sorry to say, has long since disappeared, unless, indeed, it has been preserved by subsequent publication in the Register, a point upon which I am uninformed, though, at this same meeting, Drs. Cook, Rogers, and James Taylor were appointed a "Publishing Committee," probably for the purpose of printing this and an address by Dr. John Allen, on "The History of Dental Science," also read before this meeting.

Thus, we have in these simple records the facts pertaining to the organization of one of the very first societies formed for the purpose of advancing and improving professional knowledge among the practitioners of dentistry, the vast influence of which could not then be computed by the most enthusiastic, but which is now evidenced by the 13,000 practitioners in the United States alone, some twenty dental colleges and universities, half that many more periodicals devoted to its interests, and, better than all, an enlightened and growing public opinion, which rewards the practitioner for the many hard licks he has put in.

The newly-born Society continued to grow from year to year, adding to its usefulness and membership, and exercising a harmonizing influence upon the many diverse factors which then constituted the bulk of the profession.

For instance, before the completion of the organization, while waiting for a draft of the Constitution to be made, "several communications were then made, relating principally to the malpractice of filling teeth with mineral paste."

From this, it would seem that there then existed considerable manipulative ability, and that they were afraid the mineral paste (amalgam?) would lead to a reduction of professional dignity and the size of the fee for performing a legitimate operation.

Again, in 1845, Dr. Challen offered the following resolution: "That the discoveries and improvements known to the members of the dental profession are and ought to be considered as the common property of the profession."

In the following year Dr. Wm. B. Ross delivered an address upon the propriety of patenting new remedies and discoveries in the profession. Dr. Edward Taylor then presented a resolution to the effect "That any member of this Society who may patent any instrument or mode of practice may be subject to expulsion from the Society." During the discusson which followed, Dr. James Taylor said : " No letters patent should be thrown around her (science's) pinions. Naught but the dictum of the Great Almighty should be interposed to say ' thus far shalt thou go, and no farther."

At the same meeting a resolution was adopted providing " that a committee of three be appointed by the Chair to investigate and report whether Dr. Allen, of our association, is the author of a dental improvement for which he has obtained a patent."

These instances I give to show how high the standard of professional requirement and etiquette was placed immediately upon the organization of the Society.

It also set its foot upon the universal and prevailing custom of dentists to hold as secret and inviolable any information or "trick" of which they might be possessed, to the detriment of the progress of the profession they had banded themselves together to further.

This spirit was in the right direction, and has been the means of building up that fraternal feeling which is now so characteristic of our colleges, our society meetings, and our literature.

The Society, at a very early period, made its influence widely felt throughout the medical profession, many members of which were elected to honorary membership, and greatly esteemed the favor.

Prominent among them was Professor Daniel Drake, one of the most gifted intellects the medical profession of this country has ever produced, and so high was his estimation of the scientific ability of the gentlemen composing the Society at this time, that he sought information from them to incorporate in a work on medicine he was then preparing. His letter is as follows:

*To the President of the Mississippi Valley Association of Dental Surgeons:*

SIR:—In the Historical and Practical Treatise on Our Diseases, which I am now engaged in preparing for the press, I have appropriated a chapter to maladies of the teeth, and am anxious to obtain from gentlemen devoted to their treatment, as much information as possible. Permit me, then, to request of the members of the association over which you preside, such facts and observations as they may be able to communicate on the following points:

1. What is the nature of that diathesis, or constitutional predisposition or disorder (if any), which so often occasions decay in the deciduous teeth of our children?

2. To what causes, external or pathological, local or constitutional, shall we ascribe the premature decay of the second teeth, in the West? Is a hereditary scrofulous diathesis a cause of infirm teeth? Is dyspepsia a cause of early decay? Does the acid thrown up by many dyspeptics, in paroxysms of that disease, act chemically on the teeth? What is the effect of repeated salivations on the teeth and gums? What are the effects of tobacco on the teeth, and are those of chewing and smoking the same?

3. Has the tartar of the teeth a constitutional origin?

4. Is the decay of teeth greater in the West than in the Atlantic States, in the same latitudes, and is there any difference in different latitudes in the same meridians?

5. Is there any difference as to soundness of teeth between our native and foreign population?

6. Are the teeth of our colored people less subject to decay than those of white who labor and live in a simple manner as to diet and drinks?

Replies to these questions (or information not referred to in them), on diseases of the mouth generally, communicated to me within the next few months, will be acknowledged as a favor, while I shall scrupulously give with every new or important observation the name of its author.

I have the honor to be, very respectfully, your obedient servant. DANIEL DRAKE.

This letter was read to the Society, and a resolution passed appointing a committee to comply with the request as far as possible, and also thanking Professor Drake for the interest he manifested in the promotion of dental science.

It may be mentioned in this connection that these very questions, in one form or another, have worried the brains of the profession from that day to this, and, it is safe to say, have not been adequately and scientifically answered. The man who can do so, may rest assured that his name will descend in perpetual honor in the annals of dentistry, and the people will rise up and call him blest.

Professor Drake was one of the most prominent practitioners and teachers of medicine the West has ever produced, and it was especially in Cincinnati that he displayed his powers and influence in the advancement of medical and kindred sciences. Our present famous hospital is indebted to him for its establishment, and to him also the Ohio Medical College owes a great deal of its reputation in the early days of this city. Quite recently, one of the papers on the early history of the Ohio Valley, by Prof. Venable, and published in the Cincinnati *Commercial Gazette*, gave a detailed account of his labors in Cincinnati that is not only interesting to those in this part of the country, but is of historical value.

At this meeting, 1846, was conceived the idea of publishing a dental journal and the REGISTER is the outcome of a resolution by Dr. Edward Taylor, to the effect "that a committee of three be appointed by the chair to ascertain the cost of publishing a quarterly journal devoted to the interests of our profession, and, if they think it advisable, to propose a scheme for said publication, and submit it to the next annual meeting of this society."

The committee appointed to discharge this important trust consisted of Drs. James Taylor, John Allen, and Henry Crane, and right well did they execute the commission, for the result of their labors—THE DENTAL REGISTER—is known wherever there is a practicing dentist.

At the evening session of the same day, Dr. Edward Taylor offered an amendment to his resolution, authorizing the committee to issue during the year a specimen number, not to cost in excess of $50.00.

It is, then, to Edward Taylor, that the profession owes the REGISTER, and not his brother James, as has generally been thought, though the latter was its first editor, and lent to it the energies of an active and well-stored mind.

So far as the formative period of the Ohio Dental College is concerned, my search through the early minutes of the association has been in vain, for the only mention I find of it is, that the meeting of 1846 took place in its lecture room. This, however, may be accounted for by the fact that the society as a body took no action towards its founding, though its members individually were mainly instrumental in securing its charter and the necessary funds with which to purchase the ground and erect the college building.

All these facts were, at my request, gathered together by Prof. James Taylor and presented before the Alumni Association, at its meeting at the Burnet House, this city, in 1879, and his manuscript, which had been lost sight of subsequent to his death, has since been recovered and is now in possession of the Committee on History.

So long as our association exists it may point with pride to its record of usefulness in achieving the two great feats of having been the progenitor of the oldest existing dental periodical in the world and the second oldest dental college, both of which make their influence felt all over the civilized globe.

And now a word or two concerning the place of yearly meeting. During the first five years of its life the association was accustomed to meet in Cincinnati. This habit, however, became of a migratory nature when in September of 1849 the members assembled in Louisville, deeming a change of place an advantage to the attendance, the name of the association also implying a more cosmopolitan character. In 1850 the meeting again took place in Cincinnati, returning to Louisville the following year. In 1874 the society went to St. Louis, where it held its sessions together with the Missouri State society.

This scheme did not produce the results anticipated, for these are the only instances in which the annual meeting was held at any other locality than Cincinnati.

The examination of the minutes necessary for the preparation of this sketch has brought to light the fact that the records of the association are by no means complete. Up to and including 1859 the minutes are intact; part of 1860 is missing. In 1862 no session was held, owing to the civil war. Three years' minutes are missing entirely, viz., 1866, 1868, 1869, while half of 1871 is absent.

This is certainly very much to be deplored, for it detracts from the value of the work as a continuous history of the progress of our profession during the last half century. It is barely possible that the lost records might be recovered if those taking part during the years mentioned would make inquiries of the officers then serving, and requesting that they hunt through their private papers, for there is no telling by what chance the rough draft of the proceedings may have slipped into some nook or crevice, and have remained unseen, unnoticed, all these years.

During its long existence, the association did not always have bright skies and plain sailing, as one might suppose, judging from the harmony and good feeling that have so long held sway.

In one year, 1852, things were at a low ebb, interest semed to flag, and indications pointed towards an adjournment *sine die*, and allow the College Association to become the medium of professional progress.

Upon this question, however, the members were equally divided, and the proposition was to disband and concentrate upon the above-mentioned body. In order to prevent such a calamity one of the younger and most active members hustled around and found two new members, viz., Dr. George Watt and Dr. John G. Hamill. The accession of these gentlemen evened things up, and it was the vote of the latter which turned the tide in favor of continuing the society. Interest was thus aroused anew, which resulted in increased attendance and harder work upon the part of the members in the succeeding year.

The society had many fathers, but in its hour of trial and sore distress it found its saviour in Dr. Hamill. He has long since gone to "that undiscovered country from whose bourne no traveller returns," and it is but just that those who have succeeded him should pay this tribute to his memory.

Many instances of an interesting nature crop out here and there in the pages of the records, but which it is beyond the province of this paper to treat, though I may be pardoned if I engage your attention for a moment or two longer to mention a few of them.

During the first years of the society a large number of honorary members were elected, generally, gentlemen from the medical profession. At its first meeting, it placed upon this list, Chapin A. Harris, the father of dental education, and, as Kingsley said in his address before the New England Dental Society: "Fortunate it was for posterity that Chapin A. Harris and his colleagues were denied admission to the medical colleges. They builded wiser than they knew. Dentistry, independent, has grown with a vigor unparalleled."

This is a fact to the truth of which there are living witnesses who were present at this meeting and cast their ballots in favor of Dr. Harris, who sought to affiliate himself with what he intuitively felt would be a great power in moulding the future character of the newly born profession.

In the following year, among others, three gentlemen were elected to honorary membership, whose names were then famous in their professions and which time has only served to firmly cement in the annals of science. I refer to Prof. John Locke, Prof. R. D. Mussey, and Prof. Shotwell. The first was a chemist and philosopher of national reputation, who, I believe, was the inventor and maker of the famous clock, which he spent many years in perfecting and which the United States Congress purchased for a large sum; it is now in daily use in the House of Representatives at Washington.

Prof. Mussey was the first to discover that the skin is an absorbent and that medication is possible by inunction.

Prof. Shotwell was a medical genius and was intimately identified with the early history of the Ohio Medical College.

The three Drs. Judkins were also members on the honorary list, J. P., William, and David, all of them prominent citizens and practitioners in Cincinnati half a century ago.

I might name Geo. Mendenhall, Prof. Gross, Samuel Martin and others among the most distinguished who were admitted to the councils of our dear old Society.

When I think of these men and what they were, it makes me blush to have to urge by personal appeal the attendance of members who should feel proud to be honored by having their names upon the Society's roster. The walls of this room still ring with the sound of their voices and the words of wisdom which fell from their lips reverberate from the Alleghenies to the Rockies, for is not that the Mississippi Valley ?

Of the number present at the organization, I know of but four who survive, Dr. John Allen, of New York, Dr. Charles Bonsall, Wm. M. Hunter, and A. Berry, all of Cincinnati.

It is possible that others may still be living. I hope they are and that all their days may be passed in peace and plenty.

The last one to pass away was our venerable friend Dr. J. P. Ulrey, of Lawrenceburg, Ind., his demise taking place but two or three months ago.

And now, fellow members, I trust I have not imposed too much upon your patience in presenting this incomplete outline of our Society's history, and in conclusion allow me to express the hope that we will, one and all, from this time forth, emulate the example of the honored ones gone before us.

